# Recovering 3D Shape with Absolute Size from Endoscope Images Using RBF Neural Network

**DOI:** 10.1155/2015/109804

**Published:** 2015-04-09

**Authors:** Seiya Tsuda, Yuji Iwahori, M. K. Bhuyan, Robert J. Woodham, Kunio Kasugai

**Affiliations:** ^1^Department of Computer Science, Chubu University, 1200 Matsumotocho, Kasugai 487-8501, Japan; ^2^Department of Electronics and Electrical Engineering, IIT Guwahati, Guwahati 781039, India; ^3^Department of Computer Science, University of British Columbia, Vancouver, BC, Canada V6T 1Z4; ^4^Department of Gastroenterology, Aichi Medical University, 1-1 Karimata, Yazako, Nagakute 480-1195, Japan

## Abstract

Medical diagnosis judges the status of polyp from the size and the 3D shape of the polyp from its medical endoscope image. However the medical doctor judges the status empirically from the endoscope image and more accurate 3D shape recovery from its 2D image has been demanded to support this judgment. As a method to recover 3D shape with high speed, VBW (Vogel-Breuß-Weickert) model is proposed to recover 3D shape under the condition of point light source illumination and perspective projection. However, VBW model recovers the relative shape but there is a problem that the shape cannot be recovered with the exact size. Here, shape modification is introduced to recover the exact shape with modification from that with VBW model. RBF-NN is introduced for the mapping between input and output. Input is given as the output of gradient parameters of VBW model for the generated sphere. Output is given as the true gradient parameters of true values of the generated sphere. Learning mapping with NN can modify the gradient and the depth can be recovered according to the modified gradient parameters. Performance of the proposed approach is confirmed via computer simulation and real experiment.

## 1. Introduction

Endoscopy allows medical practitioners to observe the interior of hollow organs and other body cavities in a minimally invasive way. Sometimes, diagnosis requires assessment of the 3D shape of the observed tissue. For example, the pathological condition of a polyp often is related to its geometrical shape. Medicine is an important area of application of computer vision technology. Specialized endoscopes with a laser light beam head [1] or with two cameras mounted in the head [2] have been developed. Many approaches are based on stereo vision [3]; however, the size of endoscope becomes large and this imposes a burden on the patient. Here, we consider a general purpose endoscope, of the sort still most widely used in medical practice.

Here, shape from endoscope image is considered. Shape from shading (SFS) [4] and Fast Marching Method [5] based SFS approach [6] are proposed. These approaches use orthographic projection, while an extension of FMM to the perspective projection is proposed in [7] or further extension of FMM to both point light source illumination and perspective projection is proposed in [8]. Recent extensions include generating Lambertian image from the original multiple color images [9, 10]. Application of FMM includes the solution [11] to the oblique light source problem using neural network learning [12].

Iwahori et al. [13] developed Radial Basis Function Neural Network (RBF-NN) photometric stereo, where RBF-NN is powerful to achieve the multiple dimensional nonparametric functional approximation between input and output mapping.

Recently, VBW model [14], which is based on solving the Hamilton-Jacobi equation, has been proposed to recover a shape from an image taken under the conditions of point light source illumination and perspective projection. However the result recovered by VBW model is relative and there is a problem that VBW gives much smaller values of surface gradient and height distribution than those of true values. That is, it is impossible to apply the VBW model to obtain the exact shape and size.

This paper proposes a new approach to recover the 3D shape with absolute size from 2D image taken under the condition of both point light source illumination and perspective projection. While the VBW model approach can recover the relative shape with relative scale, the proposed approach obtains absolute depth by improving the gradient modification by RBF neural network. The final purpose of this approach is to support the medical diagnosis of the status of polyp if polyp is benign or malignant by recovering 3D shape with its absolute size.

The proposed approach generates a Lambertian sphere model. VBW model is applied for the generated sphere and shape is recovered. Here RBF-NN is used and learned with this sphere to improve the accuracy of recovered shape, where input and output of the neural network are the surface gradient parameters obtained via VBW model as input and the corresponding true values as output, respectively.

The proposed approach is evaluated and it is confirmed that the obtained shape is improved via computer simulation and real experiments.

## 2. VBW Model

VBW model [14] is proposed as a model to calculate the depth from the view point under the conditions of point light source illumination and perspective projection by solving the Hamilton-Jacobi equations [15] combined with the model of Faugeras and Prados models [16, 17]. Lambertian reflectance is assumed for a target object as another condition.

The following processing is applied for each point of the image. First, the initial value for the depth *Z*_default_ is given using (1) as in [18]:

(1) $$\begin{array}{c} Z_{\text{default}}=-0.5\log\left(If^{2}\right), \end{array}$$

where *I* represents the normalized image intensity and *f* is the focal length of the lens.

Next, the combination of gradient parameters which gives the minimum gradient is selected from the difference of the depth for the neighboring points. The depth *Z* is calculated from (2) and the process is repeated until *Z* does not change for that at the previous stage. Here, (*x*, *y*) represent the image coordinates, Δ*t* represents the width of time, (*m*, *n*) represent the minimum gradient for (*x*, *y*) directions, and $Q=f/\sqrt{x^{2}+y^{2}+f^{2}}$ represents the coefficient of the perspective projection, respectively:

(2) Zx,y =Z(x,y)+Δtexp⁡(−2Z(x,y))  −Δt  ·If2Qf2mx2+ny2+xmx+yny2+Q2.

Here, it is noted that the shape obtained via VBW model gives the relative scale, not absolute one. This means that obtained result gives the smaller values of surface gradients than those actual values.

## 3. Proposed Approach

### 3.1. NN Learning for Modification of Surface Gradient

When uniform Lambertian reflectance is assumed, the intensity depends on the dot product of surface normal vector and light source vector with the inverse square law for illuminance. The image intensity of the surface is determined as follows:

(3) $$\begin{array}{c} E=C\frac{\left(s\cdot n\right)}{r^{2}}, \end{array}$$

where *E* is image intensity, *C* is reflectance parameter, **s** is a unit vector towards a point light source, **n** is a unit surface normal vector, and *r* is the distance between a point light source and surface point.

The basic assumption is that both of point light source and center of lens are located at the origin of (*X*, *Y*, *Z*) coordinates and image projection is perspective projection. That is, the object is viewed and illuminated from the view point. Here, the actual endoscope image has the color textures and specular reflectance. Using the approach proposed in the paper [19] can convert the original input image into the uniform Lambertian gray scale image.

VBW model gives the relative result for the true size and shape. VBW model also assumes the condition that Lambertian image is used to recover the shape as a target. The result gives the small values of surface gradient and the depth. Here, the modification of surface gradient and improvement of the recovered shape are considered. First the surface gradient at each point is modified with neural network (NN), and then the depth is modified from modified surface gradient parameters (*p*, *q*) = (∂*Z*/∂*X*, ∂*Z*/∂*Y*). RBF-NN (Radial Basis Function Neural Network) [12] is used for the learning for modification of surface gradient of the result obtained by VBW model.

Expanding (3) with parameters (*p*, *q*, *Z*) derives the following:

(4) $$\begin{array}{c} E=C\frac{\left(-px-qy+f\right)f^{2}}{{\left(x^{2}+y^{2}+f^{2}\right)}^{3/2}Z^{2}{\left(p^{2}+q^{2}+1\right)}^{1/2}}, \end{array}$$

where (*x*, *y*) are image coordinates, *f* is focal length of the lens, and *Z* is depth.

**Figure 1:**
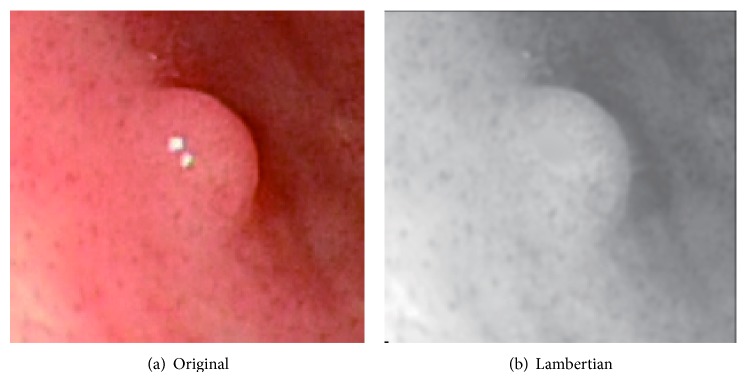
Endoscope image and Lambertian image.

Sphere image is synthesized using (4) and VBW model is applied to this sphere image. Surface gradient parameters (*p*, *q*) are obtained using forward difference of *Z* obtained from VBW model. Calculated (*p*, *q*) and the corresponding true (*p*, *q*) for the synthesized sphere are given to the RBF-NN as input vector and output vector, respectively, and NN learning is applied. After NN learning, this NN can be used to modify the recovered shape for other images. Original endoscope image is shown in Figure 1(a) and generated Lambertian image using [19] is shown in Figure 1(b) as an example.

**Figure 2:**
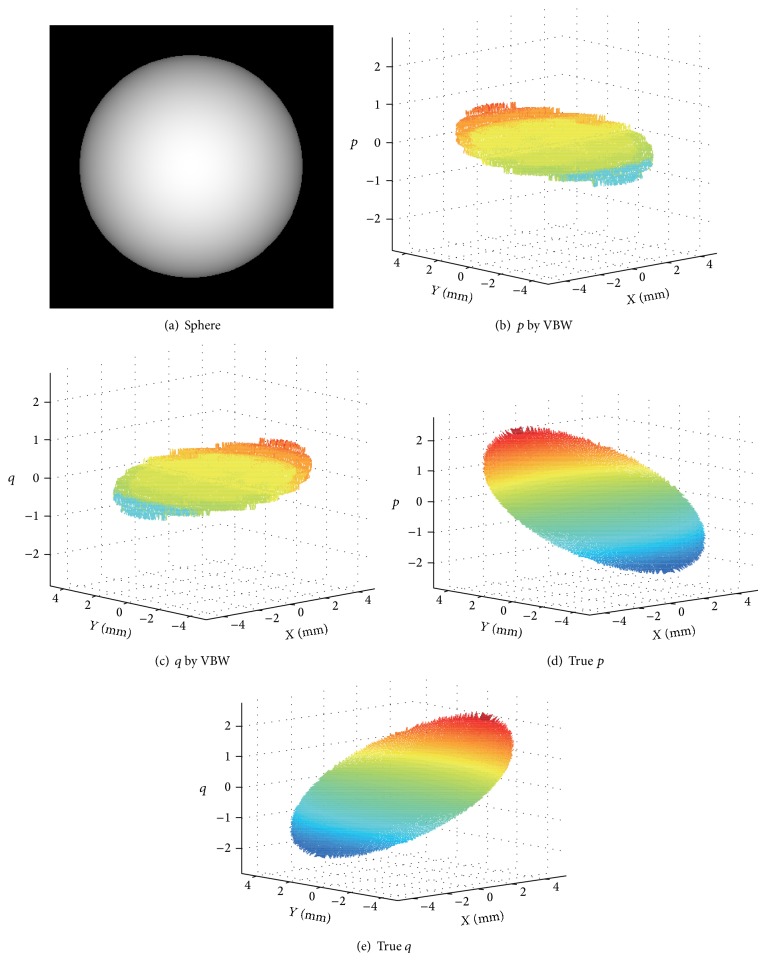
Synthesized sphere for NN learning.

**Figure 3:**
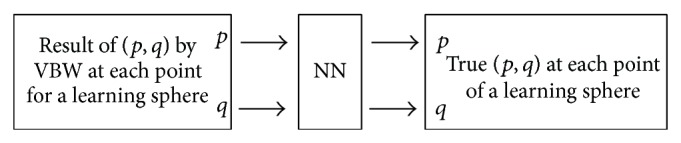
Learning flow.

The synthesized sphere image used in NN is shown in Figure 2(a). Surface gradients obtained by VBW model are shown in Figures 2(b) and 2(c) and the corresponding true (*p*, *q*) of this sphere are shown in Figures 2(d) and 2(e), respectively. Various points are sampled from a sphere and NN learning is done except points with so large values of (*p*, *q*). Procedure of NN learning is shown in Figure 3.

### 3.2. NN Generalization and Modification of *Z*

Learned NN is used for generalization for another test object. Modification of (*p*, *q*) using learned NN is applied to test object and depth *Z* is calculated and updated using modified (*p*, *q*). To apply this NN to endoscope image, specular component is removed and uniform Lambertian image is generated based on our previous preprocessing for endoscope image in [19]. This is because endoscope image includes color textures and specular reflectance components and it is necessary to generate a uniform Lambertian sphere with gray scale image.

Next, VBW model is applied to this Lambertian image and (*p*, *q*) are calculated from the obtained *Z* distribution. Calculated (*p*, *q*) are input to the learned NN and the modified (*p*, *q*) are obtained as output of NN. The depth *Z* is calculated and updated by (5) using modified (*p*, *q*), where (5) is also the original equation derived in [8]:

(5) $$\begin{array}{c} Z=\sqrt{\frac{CV\left(-px-qy+f\right)}{E{\left(p^{2}+q^{2}+1\right)}^{1/2}}}, \end{array}$$

where *p*, *q*, *E*, *f*, and *C* are the same parameters as those in (4), while *V* = *f* ^2^/(*x* ^2^ + *y* ^2^ + *f* ^2^)^3/2^.

**Figure 4:**
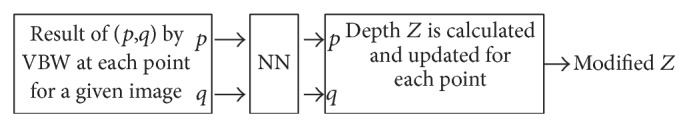
Flow of NN generalization.

The flow of processing described above is shown in Figure 4.

## 4. Experimental Results

### 4.1. NN Learning

**Figure 5:**
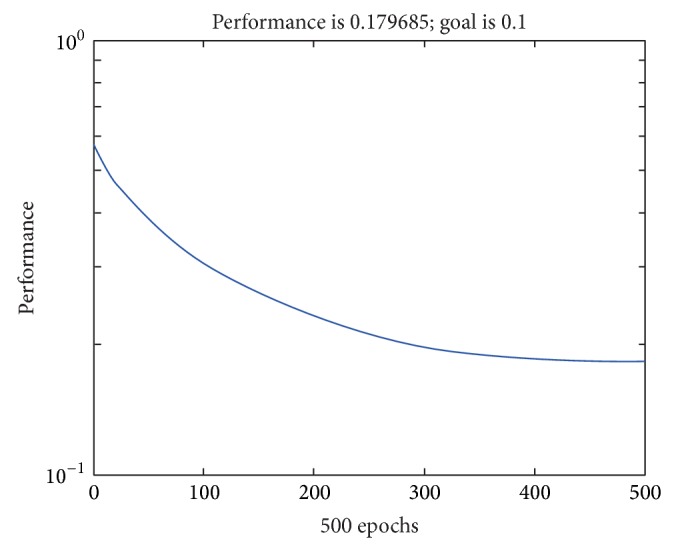
Learning result.

Sphere was synthesized with radius 5 mm whose center is located at (0, 0, 15) with the focal length 10 mm of the lens and reflectance parameter set to 100. The image size is 9 mm × 9 mm and pixel size is 256 × 256 pixels. This sphere was recovered by VBW model and the result gave the gradient parameters (*p*, *q*) as shown in Figures 2(b) and 2(c), respectively. These (*p*, *q*) are used as input of NN and the corresponding true (*p*, *q*) shown in Figures 2(d) and 2(e) are used as output of NN. Learning was done under the condition of the error goal 1.0*e* − 1, the maximum number of learning epochs 500. The results of learning are shown in Figure 5.

As shown in Figure 5, NN learning was done with 500 epochs. Also, processing time for NN learning was around 70 seconds.

A sphere has a variety of surface gradients and it is used for the NN learning. After a sphere is used for NN learning, not only a sphere object but also another object with another shape including convex or concave surfaces is also applied in the generalization process. This is because surface gradient for each point is modified by NN and this modification does not depend on the shape of target object.

### 4.2. Computer Simulation

**Figure 6:**
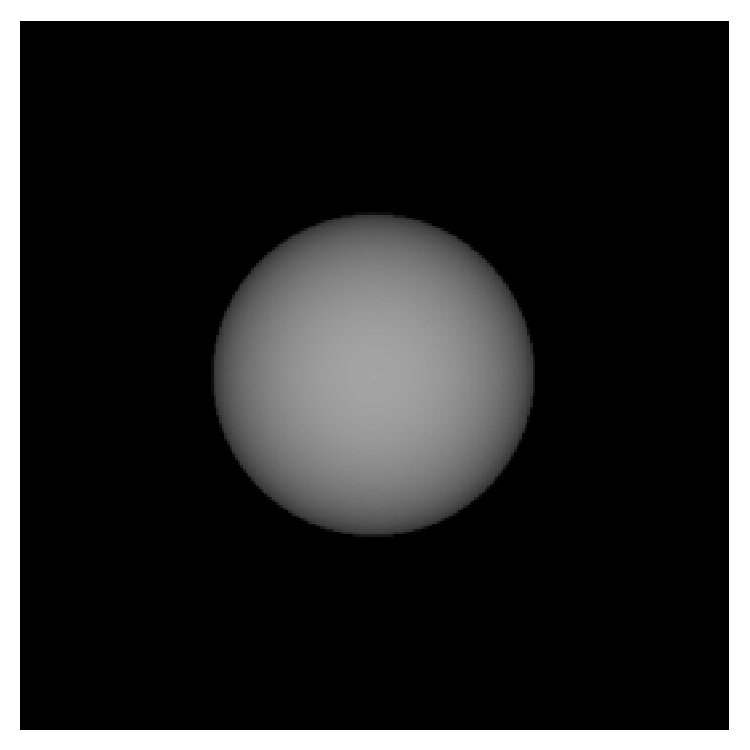
Sphere image.

**Figure 7:**
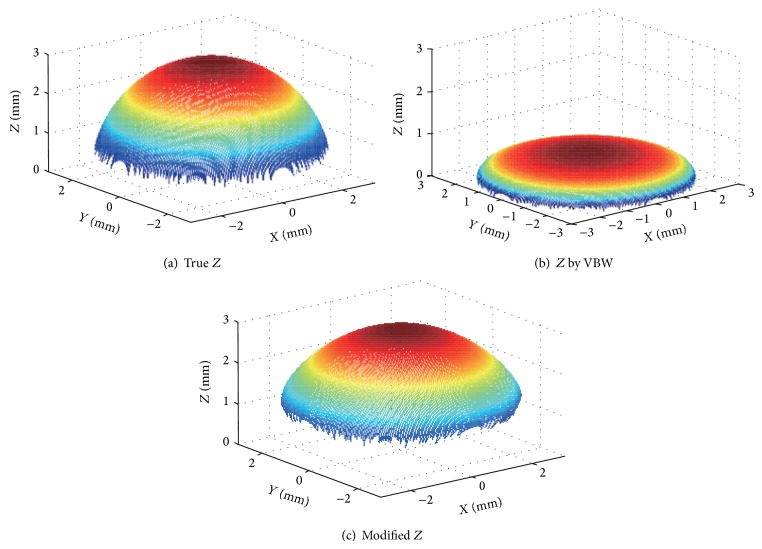
Results.

Computer simulation is done to confirm the performance of NN generalization. The first experiment is done under the condition that the reflectance factor is 50 and the focal length is 10 mm for a sphere with radius 3 mm. The center of a sphere is set at (0, 0, 15), as shown in Figure 6. The image size is 9 mm × 9 mm and the pixel size is 360 × 360 pixels. True depth is shown in Figure 7(a) and the result of VBW model is shown in Figure 7(b), while the improved result is shown in Figure 7(c). The mean error of gradient parameters and depth is shown in Table 1.

The mean errors of surface gradient and depth are shown in Table 1. In Table 1, the depth had improvement of the mean error from 1.84 to 0.03; that is, mean error became 0.02 times less in comparison with that by VBW model. Generalization of NN was quite good for the different condition of *Z* with another size and shape. It took 40 seconds in total. It is shown that the obtained result was improved from Figures 7(a), 7(b), and 7(c) and Table 1. These results suggest that error tends to increase at the points where the values of (*p*, *q*) become large, because the number of sampled points with the larger values of (*p*, *q*) was smaller using every equal number of dot sampling.

**Figure 8:**
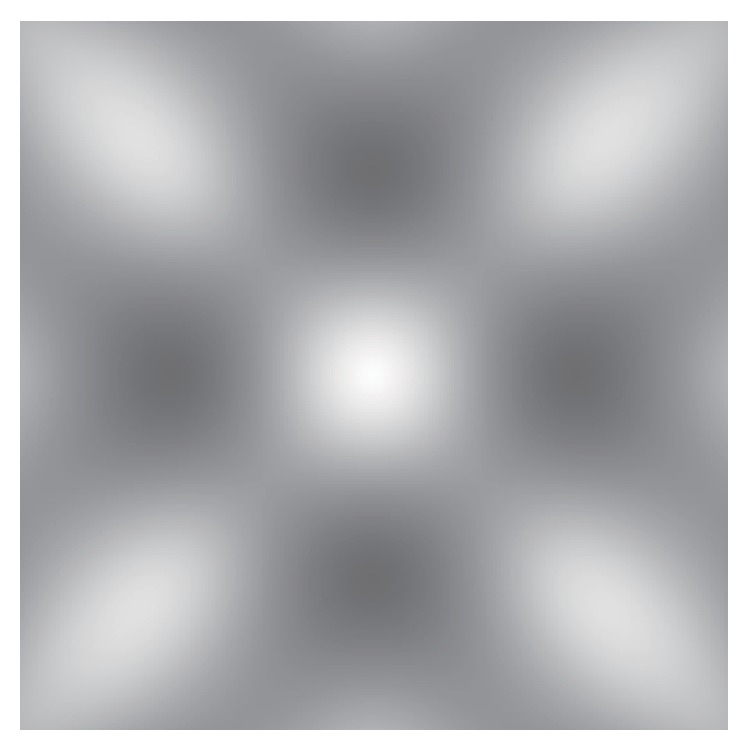
Cosine model.

Next, synthesized cosine curved surface was used, whose center is located at the coordinate (0, 0, 12). Here, the reflectance parameter *C* is 120, the focal length *f* is 10 mm, waveform cycle is 4 mm, and ± amplitude is 1 mm. Synthesized image is shown in Figure 8.

**Figure 9:**
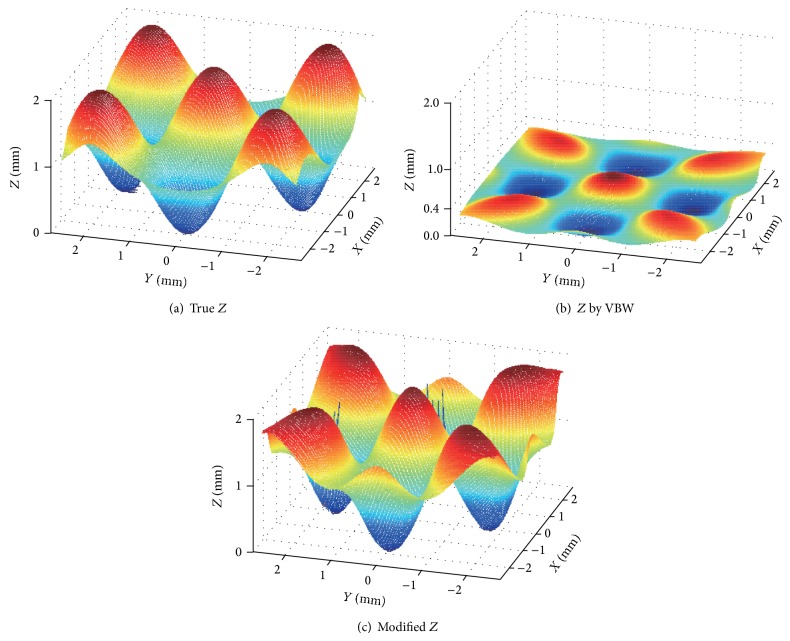
Results.

Using the learned NN, (*p*, *q*) obtained from VBW were input and generalized. (*p*, *q*) were modified and *Z* was further updated using (5). The true depth is shown in Figure 9(a). Recovered result by VBW is shown in Figure 9(b) for Figure 8 and modified depth using NN and (5) is shown in Figure 9(c).

The mean errors of surface gradient and depth are shown in Table 2. In Table 2, the depth had improvement of the mean error from 0.86 to 0.26; that is, mean error became 0.3 times less in comparison with that by VBW model. Generalization of NN was quite good for the different condition of *Z* with another size and shape. It took 9 seconds to recover the shape while it took 61 seconds for NN learning with 428 learning epochs; that is, it took 70 seconds in total.

It is confirmed that the shape is improved with modification in the proposed approach from Figure 9. This means that NN modified (*p*, *q*) for each point and *Z* are modified correctly.

### 4.3. Real Image Experiments

**Figure 10:**
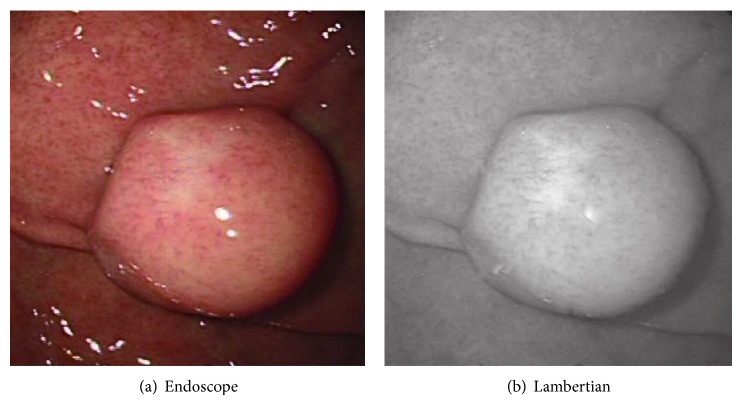
Endoscope image and generating Lambertian image.

**Figure 11:**
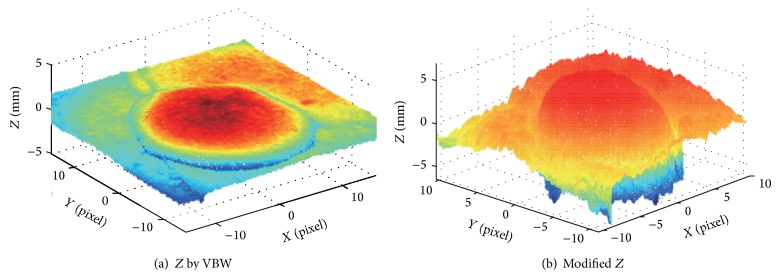
Result for endoscope images.

Real endoscope image is used in the experiments. First, NN was learned using a synthesized sphere as well. Then, VBW is applied to real endoscope image which is converted into uniform Lambertian image. Surface gradients (*p*, *q*) were modified with NN; then *Z* was calculated and updated for each point of endoscope image, where the focal length *f*, the image size, and camera movement Δ*Z* were assigned to the same known parameters as those in the computer simulation. Endoscope image is shown in Figure 10(a). Generated Lambertian image is shown in Figure 10(b). Result by VBW is shown in Figure 11(a) and modified result is shown in Figure 11(b).

In Figure 10(b), the specular reflection component was removed in comparison with Figure 10(a) of input image, and it is confirmed that the converted image has become a gray scale image with uniform reflectance. It is also confirmed that Figure 11(b) gives larger height than Figure 11(a), via modification. Except the cast shadow region, the processing was done correctly and improved the depth. The size of polyp was 1 cm and the processing time for shape modification was 9 seconds. It took 9 seconds to recover the shape while it took 117 seconds for NN learning with 540 learning epochs; that is, it took 126 seconds in total. Although the quantitative evaluation is difficult, medical doctors with experience of endoscope diagnosis evaluated the result and qualitatively correct evaluations have been obtained for the result. Thus, it was confirmed that the proposed approach is effective for the real endoscope image.

**Figure 12:**
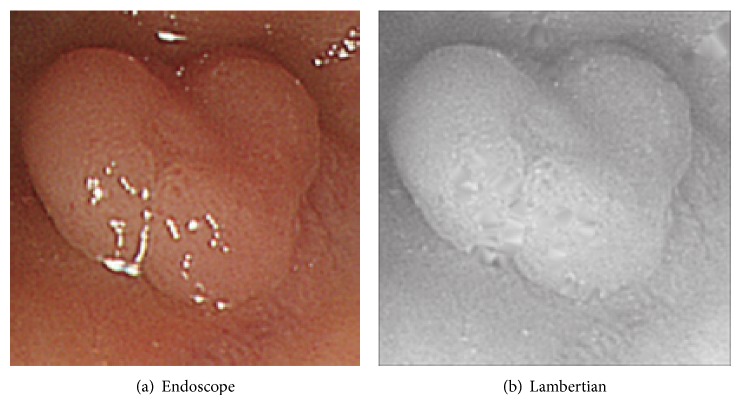
Endoscope image and generating Lambertian image.

**Figure 13:**
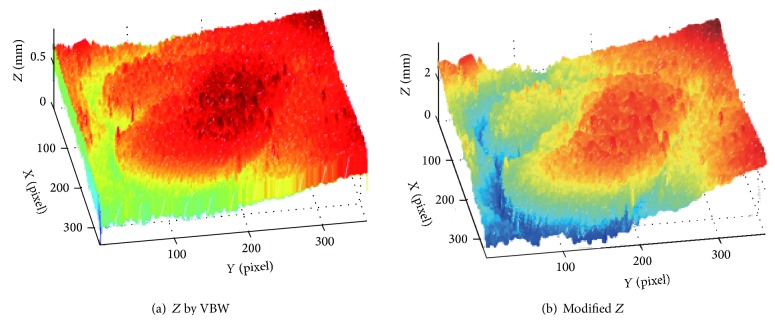
Result for endoscope images.

Another experiment is done for three cases of endoscopic image. Endoscope image of the first case is shown in Figure 12(a), and this Lambertian image generated is in Figure 12(b). The result for Figure 12(b) is shown in Figure 13(a), while that for the proposed approach is shown in Figure 13(b), respectively.

**Figure 14:**
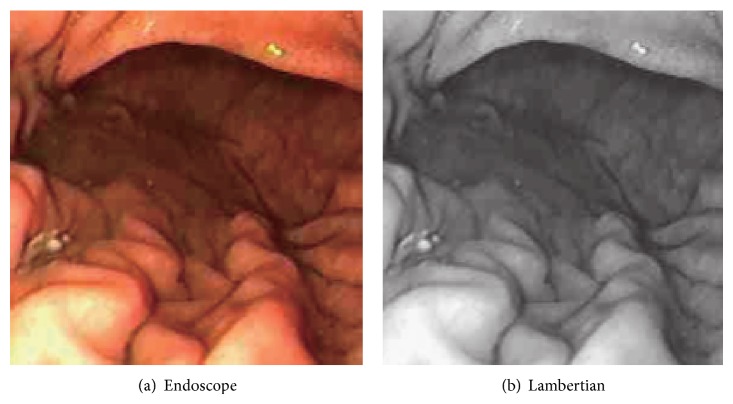
Endoscope image and generating Lambertian image.

**Figure 15:**
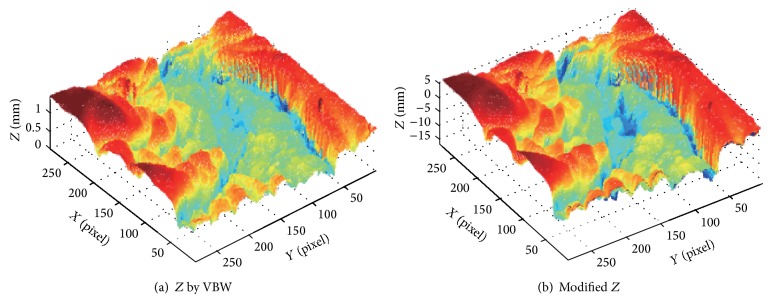
Result for endoscope images.

Endoscope image of second case is shown in Figure 14(a), and this Lambertian image generated is in Figure 14(b). The result for Figure 14(b) is shown in Figure 15(a), while that for the proposed approach is shown in Figure 15(b), respectively.

**Figure 16:**
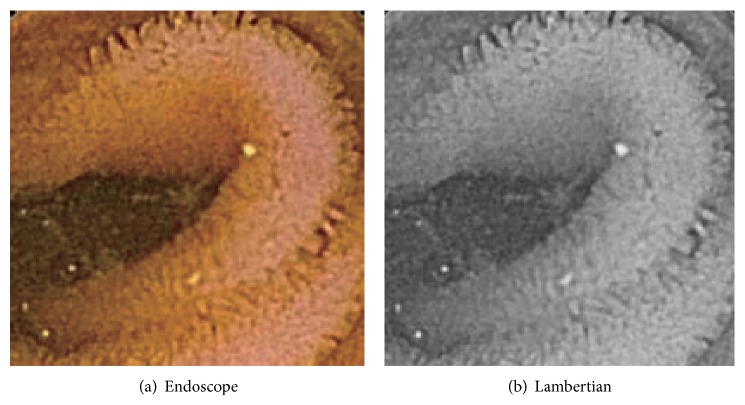
Endoscope image and generating Lambertian image.

**Figure 17:**
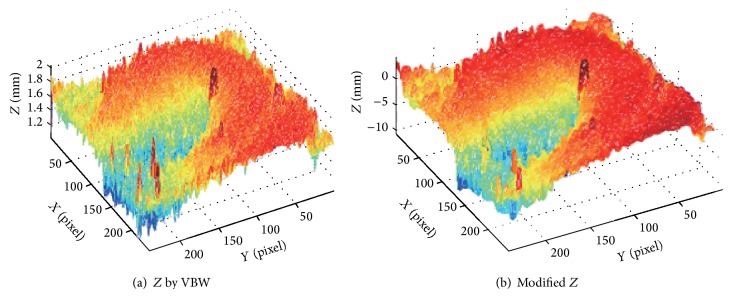
Result for endoscope images.

Endoscope image of third case is shown in Figure 16(a), and this Lambertian image generated is in Figure 16(b). The result for Figure 16(b) is shown in Figure 17(a), while that for the proposed approach is shown in Figure 17(b), respectively.

In Figure 13(b), the size of polyp was 2 mm. In Figures 12(b), 14(b), and 16(b), the specular reflection component was removed in comparison with Figures 12(a), 14(a), and 16(a) of input image, and it is confirmed that the converted images have become a gray scale images with uniform reflectance, recpectively. It is also confirmed that Figures 13(b), 15(b), and 17(b) give larger height than Figures 13(a), 15(a), and 17(a) via modification, respectively. Learning time in all examples is about 60 seconds, while it took about 10 seconds to recover the modified shape. In Figures 15(b) and 17(b), the proposed approach can recover the rough concave/convex shape. It was confirmed that the gradient modification is effective to other shapes except a sphere. The result by VBW model represents convex and concave shape with relative scale for whole examples. However the height result by VBW model gives very small height and does not represent actual height, which means the height obtained is relative. The advantage of the proposed approach is that it can recover 3D shape with absolute size of polyp by keeping the original convex and concave conditions to obtain the actual status of polyp.

## 5. Conclusion

This paper proposed a new approach to recover the 3D shape with absolute size from 2D image taken under the condition of both point light source illumination and perspective projection. While the VBW model approach can recover the relative shape with relative scale, the proposed approach obtains absolute depth by improving the gradient modification by RBF neural network. Recovering 3D shape with its absolute size proposed here makes it possible to support the medical diagnosis for the status of polyp if polyp is benign or malignant.

NN was introduced to demonstrate the modification of surface gradient using a synthesized sphere. VBW model is used to recover the original shape and further modification of accuracy of shape is performed via RBF-NN. Here, no parametric functional form has been assumed to improve the shape via NN. This has an important role in recovering the correct *Z* from the modified surface gradient. The approach is evaluated in computer simulation and real experiment using endoscope images. It was confirmed that the approach can improve the accuracy of recovered shape with acceptable error range. Other extensions of shape recovery algorithm or NN modification with whole camera parameters remain in the further subject.

## Figures and Tables

**Table 1:** Mean error.

	*p*	*q*	*Z* [mm]
VBW	11.24	11.24	1.84
Proposed	0.41	0.41	0.03

**Table 2:** Mean error.

	*p*	*q*	*Z* [mm]
VBW	23.04	23.04	0.86
Proposed	0.33	0.33	0.26
